# Repeated PM2.5 exposure inhibits BEAS-2B cell P53 expression through ROS-Akt-DNMT3B pathway-mediated promoter hypermethylation

**DOI:** 10.18632/oncotarget.7842

**Published:** 2016-03-02

**Authors:** Wei Zhou, Dongdong Tian, Jun He, Yimei Wang, Lijun Zhang, Lan Cui, Li jia, Li Zhang, Lizhong Li, Yulei Shu, Shouzhong Yu, Jun Zhao, Xiaoyan Yuan, Shuangqing Peng

**Affiliations:** ^1^ Evaluation and Research Center for Toxicology, Institute of Disease Control and Prevention, Academy of Military Medical Sciences, Beijing 100071, PR China

**Keywords:** PM2.5, Akt, DNMT3B, P53, hypermethylation

## Abstract

Long-term exposure to fine particulate matter (PM2.5) has been reported to be closely associated with the increased lung cancer risk in populations, but the mechanisms underlying PM-associated carcinogenesis are not yet clear. Previous studies have indicated that aberrant epigenetic alterations, such as genome-wide DNA hypomethylation and gene-specific DNA hypermethylation contribute to lung carcinogenesis. And silence or mutation of *P53* tumor suppressor gene is the most prevalent oncogenic driver in lung cancer development. To explore the effects of PM2.5 on global and *P53* promoter methylation changes and the mechanisms involved, we exposed human bronchial epithelial cells (BEAS-2B) to low concentrations of PM2.5 for 10 days. Our results indicated that PM2.5-induced global DNA hypomethylation was accompanied by reduced DNMT1 expression. PM2.5 also induced hypermethylation of *P53* promoter and inhibited its expression by increasing DNMT3B protein level. Furthermore, ROS-induced activation of Akt was involved in PM2.5-induced increase in DNMT3B. In conclusion, our results strongly suggest that repeated exposure to PM2.5 induces epigenetic silencing of *P53* through ROS-Akt-DNMT3B pathway-mediated promoter hypermethylation, which not only provides a possible explanation for PM-induced lung cancer, but also may help to identify specific interventions to prevent PM-induced lung carcinogenesis.

## INTRODUCTION

Particulate matter (PM), an important type of air pollutants, has been classified as a Group 1 human carcinogen by the International Agency for Research on Cancer (IARC) [1]. Growing epidemiological studies have documented an association between high concentrations of PM, especially particles with an aerodynamic diameter less than 2.5 μm (PM2.5), and increased risk of lung cancer [2]. A large prospective study reported that each 10 μg/m^3^ increase in PM2.5 concentration was associated with a 15%−27% increase in lung cancer mortality [3]. Despite the well-known association between PM2.5 exposure and lung cancer risk, the mechanisms underlying PM2.5-induced carcinogenesis are not well understood.

Multiple genetic and epigenetic alterations affecting both tumor suppressor genes and oncogenes have been implicated in the carcinogenesis and progression of lung cancer [4, 5]. Genetic abnormalities are known to play a critical role in lung carcinogenesis, and recent studies have revealed an important contribution of epigenetic aberrations such as DNA methylation in lung cancer [6, 7]. Epidemiological studies have reported that environmental pollutant such as PM2.5 may have pleiotropic effects on DNA methylation patterns, including both gene-specific hypermethylation (such as p16 and RASSF1A promoter hypermethylation) and global DNA hypomethylation [7–10], which may lead to chromosomal instability and trigger the initiation of carcinogenesis [11, 12].

The tumor suppressor P53 is a crucial mediator of cell proliferation, apoptosis, senescence, and transformation in response to cellular damage [13, 14]. Reduced expression or silencing of *P53* by promoter hypermethylation has been reported in several neoplasms, but not in lung cancer [15, 16]. However, *P53* mutation or silencing is recognized as a frequent milestone during the development of lung cancer [17]. You *et al* demonstrated about 30% of the mutations of *P53* occur on methyl cytosine bases in the 5′ sequence, possibly because methyl cytosine is more conducive to DNA adduct formation [18]. Altered methylation pattern of the *P53* gene or its promoter may thus be an early molecular event for *P53* mutation happened during the initiation of lung cancer. Furthermore, concomitant promoter methylation of other genes in lung cancer suggests that there may be a common signaling pathway responsible for altered DNA methylation.

In mammalian cells, five DNA family methyltransferases (DNMTs) have been identified: DNMT1, DNMT2, DNMT3A, DNMT3B, and DNMT3L. Among these, DNMT1, DNMT3A and DNMT3B are responsible for maintenance of whole genome methylation pattern and *de novo* DNA methylation in cells [19, 20]. Earlier studies confirmed that increased DNMT3B activity or expression play a key role in epigenetic silencing of specific genes during the early stage of lung cancinogenesis [21]. The serine/threonine kinase Akt, also known as protein kinase B or PKB, is often phosphorylated by different carcinogens in human cells and then up-regulates the expression of DNMT3B at transcriptional or post-transcriptional levels [22–24]. Previous studies have suggested that reactive oxygen species (ROS)-activated Akt participates in ultrafine carbon black (ufCB)-induced cell proliferation, and mediates inflammatory response and pro-carcinogenic effects induced by diesel exhaust particle and smoking compounds, respectively [25–27]. So, we speculated that repeated exposure to PM2.5, modeling the real-world exposure scenarios, may lead to *P53* promoter hypermethylation through the ROS-Akt-DNMT3B pathway.

## RESULTS

### PM2.5 characterization and designation of exposure concentration

**Figure 1:**
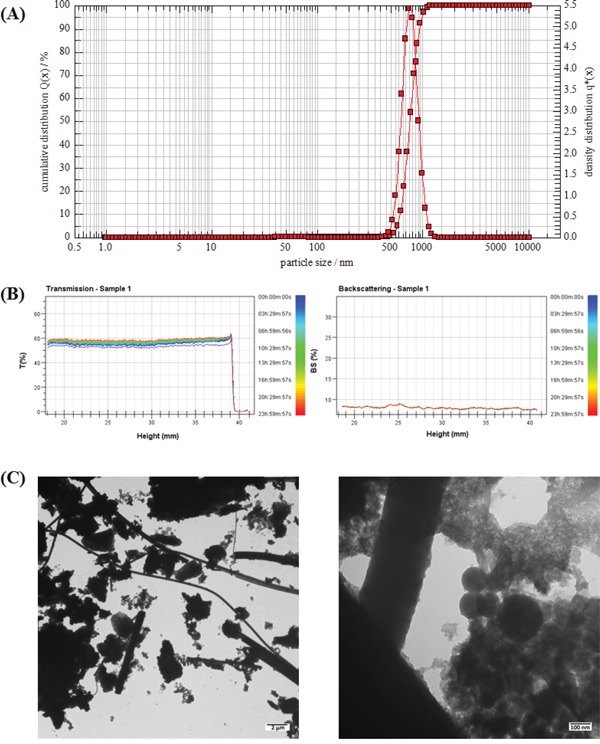
Characterization of PM2.5 **A.** The size distribution of PM2.5 in culture medium. **B.** Results of the stability analysis of PM2.5 in culture medium. **C.** The TEM images of PM2.5.

As shown in Figure 1A, the average diameter of PM2.5 in DMEM containing 2% FBS was about 0.74 ± 0.08 μm. The particles showed an approximately normal size distribution and good stability in dispersion medium (Figure 1A, 1B). TEM images revealed different shapes and sizes (Figure 1C).

The predicted concentration of PM2.5 deposited on the surface of tracheal-bronchial epithelium, as calculated by Multiple-path Particle Dosimetry (MPPD) Model software [28], was about 54.1 μg/m^2^ after a 24-h exposure at the real-world daily concentration of 120 μg/m^3^ in the north area of China in 2011. The baseline set of MPPD inputs was shown in Table S1 (in supplementary material). Given safety factors for PM2.5 exposure, we performed the repeated exposure experiments by magnifying the deposited concentration about 250-1000 times to 1.5, 3 and 6 μg/cm^2^. But we also observed glass fibers released from the filters in the particles samples (Figure 1C). Therefore, we performed the extraction method on blank filters and compared the mass extracted from blank filters to that extracted from filters with PM. We found that each 100 microgram of PM2.5 samples contained 4.12 microgram fibers. So there were only 0.247 μg/cm^2^ fibers in high dose of PM2.5 (6μg/cm^2^), which did not have significant effects on cell viability (Figure S1).

**Figure 2:**
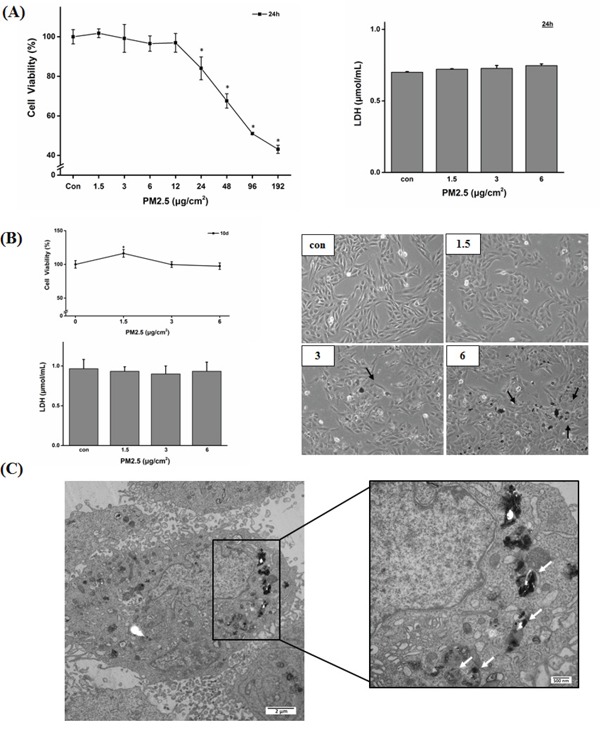
Cell viability and morphology of BEAS-2B cells after 24 h or 10 days of exposure to PM2.5 **A.** Results of cell viability and LDH release. BEAS-2B cells were treated with PM2.5 for 24 h and cell viability and cytotoxicity were detected by CCK-8 and LDH release assay (*P < 0.05 vs Con). **B.** Cytotoxicity (shown by CCK-8 and LDH release assay) and morphologic observation of BEAS-2B cells exposed to PM2.5 for 10 days. Black arrows showed settled and adherent PM2.5 to cells. **C.** Uptake of particles in BEAS-2B cell after 10 days of exposure to 6 μg/cm^2^ of PM2.5 (white arrow).

The CCK-8 and LDH release assay indicated that at these low concentrations, PM2.5 did not exert significant cytotoxicity after 24 h or 10 days of exposure (Figure 2A, 2B). Settled and adherent particles on cell surface did not affect cell morphology (Figure 2B), although there was obvious endocytosis of PM2.5 in BEAS-2B cells after 10 days of exposure to 6 μg/cm^2^ of PM2.5 (Figure 2C). Subsequent experiments were performed at these non-cytotoxic doses in order to assess the epigenetic toxic effects of PM2.5 in BEAS-2B cells.

### PM2.5 induced *P53* promoter hypermethylation, possibly via DNMT3B but not DNMT1 or DNMT3A

**Figure 3:**
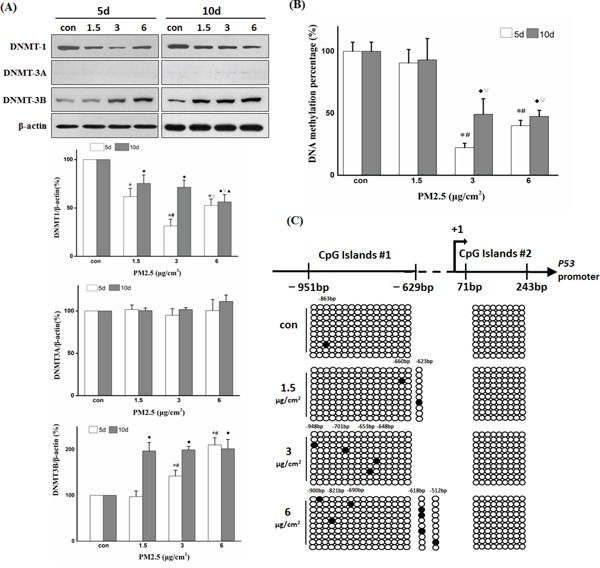
Repeated exposure to PM2.5 induced *P53* promoter hypermethylation, possibly via DNMT3B but not DNMT1 or DNMT3A **A.** Effects of repeated PM2.5 exposure on DNMTs expression determined by Western blot and quantification of DNMTs bands using the Image J software. **B.** Global DNA methylation levels after 5 or 10 days of repeated exposure to PM2.5. **C.** Methylation status of cytosine residues in *P53* promoter (−979 to +243 bp) after 10 days of exposure to 6 μg/cm^2^ of PM2.5 detected by BSP. (5d: *P < 0.05 vs Con, ^#^*P < 0.05* vs 1.5, ^◊^*P < 0.05* vs 3; 10d: ♦*P < 0.05* vs Con, ^ξ2207;^*P < 0.05* vs 1.5, ◊*P < 0.05* vs 3).

In mammalian cells, DNMT1, DNMT3A, and DNMT3B participate in both maintenance of global DNA methylation patterns and gene-specific *de novo* DNA methylation. To investigate the effect of repeated PM2.5 exposure on epigenetic regulation at relatively low concentrations, we first examined the expression changes of these three DNA methyltransferases after 5 and 10 days of PM2.5 exposure (Figure 3A). The exposure to PM2.5 significantly decreased DNMT1 expression and enhanced the expression of DNMT3B, but showed no effect on the expression of DNMT3A. Repeated PM2.5 treatment induced global loss of DNA methylation, which is normally maintained by DNMT1. The percentage of global DNA methylation decreased to half or less in 3 or 6 μg/cm^2^ group after 5 or 10 days of exposure (Figure 3B). The methylation status of *P53* promoter region was analyzed by bisulfite sequencing PCR. Remarkably, BSP results showed a progressive increase in methylated sites in *P53* promoter region with PM2.5 concentration after 10 days of treatment, and sequence analysis showed that most of the methylated bases were not located in CpG sites (Figure 3C). We also observed a positive relationship between DNMT3B expression and *P53* promoter methylation level. Taken together, these results suggest a potential role for DNMT3B (rather than DNMT1 or DNMT3A) in *P53* promoter hypermethylation.

### Promoter hypermethylation induced by PM2.5 inhibited the expression of *P53*

**Figure 4:**
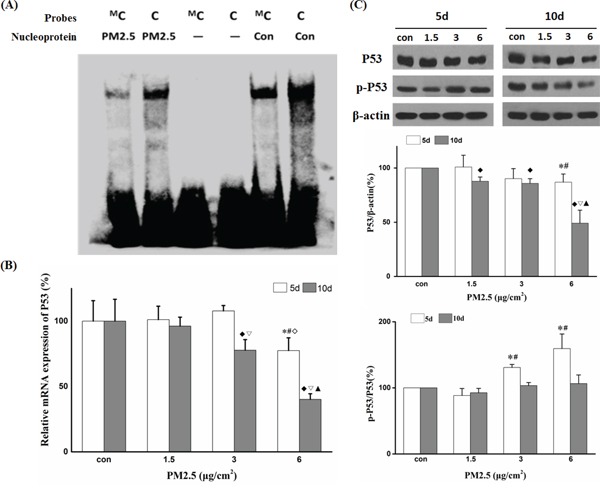
PM2.5 downregulated *P53* expression by promoter hypermethylation **A.** Differential binding of nuclear proteins to *P53* -618 methyl cytosine probe (shown as Probe^M^C) or *P53* -618 cytosine probe (shown as Probe C) detected by EMSA. **B.** *P53* mRNA expression after 5 and 10 days of repeated exposure detected by quantitative real time-polymerase chain reaction (qRT-PCR). **C.** Western blot analysis of P53 or p-P53 protein levels after 5 or 10 days of repeated exposure and quantification of protein band intensity using the Image J software. (5d: *P < 0.05 vs Con, ^#^*P < 0.05* vs 1.5, ◊*P < 0.05* vs 3; 10d: ^♦^*P < 0.05* vs Con, ^ξ2207;^*P < 0.05* vs 1.5, ^◊^*P < 0.05* vs 3).

The change in methylation pattern in promoter regions may affect gene expression by decreasing interactions with DNA-binding proteins such as transcription factors (TFs) [29]. To investigate whether *P53* promoter hypermethylation affects interactions between DNA and TFs, the hypermethylated sequences were loaded into the ALGGN website to identify potential binding sites (http://alggen.lsi.upc.es/cgi-bin/promo_v3/promo/promoinit.cgi?dirDB=TF_8.3, and maximum matrix dissimilarity rate was set at 5). The results revealed the existence of a potential binding site for C/EBP at the methyl cytosine bases in 6 μg/cm^2^ group. EMSA assay was performed to analyze the effect of methylated cytosine on the binding between DNA probes and nuclear proteins. Figure 4A demonstrated that the methylated cytosine probe decreased the binding with nuclear proteins compared to unmethylated cytosine probe, especially in 6 μg/cm^2^ group. RT-PCR and Western blot showed that the relative mRNA expression and the protein level of *P53* were significantly decreased after exposure to 6 μg/cm^2^ of PM2.5 for 10 days compared to the other treatment groups (Figure 4B and 4C). These findings suggest that transcription silencing via promoter hypermethylation (possibly catalyzed by overexpressed DNMT3B) contributes to PM2.5-induced down-regulation of *P53* expression.

### DNMT3B-siRNA reversed PM2.5-induced down-regulation of P53 expression

**Figure 5:**
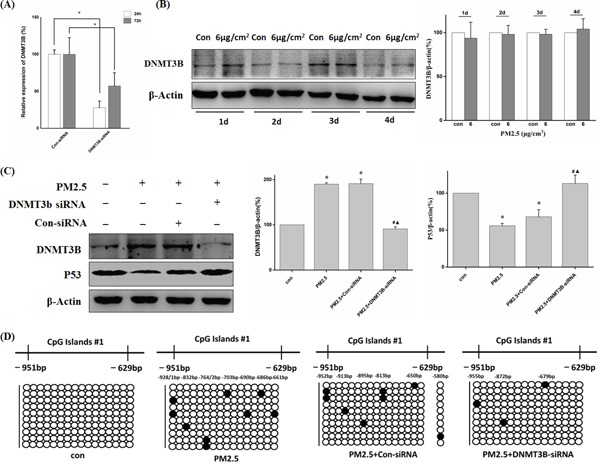
DNMT3B-siRNA reversed decreased P53 expression through down-regulation of PM2.5-induced *P53* promoter hypermethylation **A.** The gene-silencing efficiency of DNMT3B-siRNA. The mRNA expression of DNMT3B was measured by qRT-PCR after transfected with DNMT3B-siRNA for 24 h or 72 h (*P < 0.05 vs Con-siRNA). **B.** DNMT3B protein level was not increased until 5 days of exposure. **C.** Western blot analysis of DNMT3B and P53 protein levels after 10 days of PM2.5 exposure (6 μg/cm^2^) with Con-/DNMT3B-siRNA interference from day 4 to day 10, and quantification of protein band intensity normalized to β-actin (*P < 0.05 vs Con, ^#^*P < 0.05* vs PM2.5, ^◊^*P < 0.05* vs PM2.5+Con-siRNA). **D.** *P53* promoter methylation status detected by BSP.

To further examine the contribution of DNMT3B to methylation mediated-silencing of *P53*, we transiently transfected BEAS-2B cells with DNMT3B-siRNA during exposure to PM2.5 and then examined the changes in P53 expression. First, we confirmed the silencing efficiency of DNMT3B-siRNA. DNMT3B mRNA expression was reduced by 72.2% at 24 h post-transfection and remained at 57.2% at 72 h after transfection with DNMT3B-siRNA compared to control siRNA group (Figure 5A). Furthermore, PM2.5 did not exert any effect on DNMT3B protein during day 1 to day 4 (Figure 5B). Therefore, during 10 days of PM2.5 exposure, BEAS-2B cells were transiently transfected with DNMT3B or control siRNA on the 4^th^ and 7^th^ treatment day, which would substantially reduce DNMT3B expression throughout the treatment course. After 10 days of PM2.5 treatment, DNMT3B siRNA inhibited the PM2.5-induced increase in DNMT3B protein and significantly restored the expression of P53 (Figure 5C). BSP results indicated that DNMT3B-siRNA transfection decreased the amount of methylated cytosine in *P53* promoter in 6 μg/cm^2^ group (Figure 5D). These findings provide further evidence that DNMT3B mediates PM2.5-induced hypermethylation of *P53* promoter.

### PM2.5 induced akt phosphorylation through ROS generation

**Figure 6:**
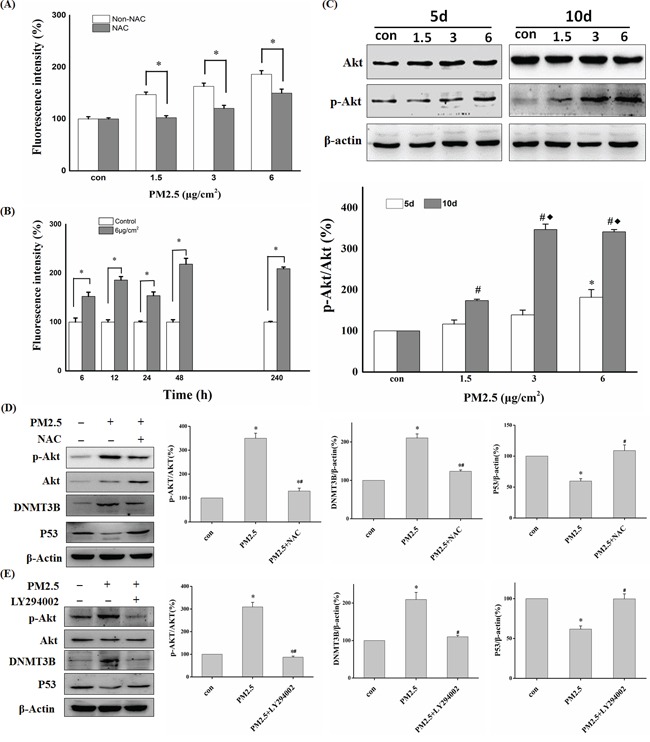
ROS and Akt participated in PM2.5-induced downregulation of P53 expression **A.** BEAS-2B cells were pretreated with or without NAC followed by PM2.5 exposure for 12 h and ROS generation was detected by Flow cytometry (*P<0.05 vs Non-NAC). **B.** ROS detection at different time points in control or 6 μg/cm^2^ group (*P<0.05 vs Con). **C.** Western blot analysis of p-Akt and Akt protein levels after 5 or 10 days of exposure to PM2.5 (5d: *P < 0.05 vs Con; 10d: ^#^*P < 0.05* vs Con, ^♦^*P < 0.05* vs 1.5). **D, E.** After pretreatment with NAC or Akt inhibitor (LY294002), the expression of p-Akt, Akt, DNMT3B, and P53 was detected by Western blot in BEAS-2B cells exposed to PM2.5 for 10 days. Quantification of protein band intensity was performed using Image J software (*P<0.05 vs Con, ^#^*P < 0.05* vs PM2.5).

It has been established that PM2.5 exposure can induce intracellular ROS generation and lead to phosphorylation activation of Akt [30, 31]. The potential of PM2.5 to induce intracellular ROS generation was detected by loading BEAS-2B cells with ROS-sensitive probe CM-H2DCFDA. The results showed that exposure to PM2.5 induced ROS generation in a dose-dependent manner (Figure 6A). Meanwhile, the ROS induced by 12-h exposure to PM2.5 was significantly decreased by pretreatment with NAC (Figure 6A). In addition, Figure 6B showed that repeated exposure to PM2.5 led to persistent high level of ROS in BEAS-2B cells. PM2.5 also increased the expression of phosphorylated Akt protein to about 1.5-fold and about 3-fold after 5 and 10 days of continuous exposure to 6 μg/cm^2^ of PM2.5, respectively (Figure 6C). PM2.5-induced Akt phosphorylation was significantly decreased in the presence of NAC (Figure 6D), strongly suggesting that the effect is mediated by ROS generation.

### ROS-Akt pathway was involved in PM2.5-induced DNMT3B up-regulation

To explore the underlying mechanisms of DNMT3B up-regulation by repeated PM2.5 exposure, we assessed the contributions of various signal pathways linked to PM2.5 exposure in previous studies. First, we assessed the phosphorylation level of JNK, ERK1/2, and Akt in BEAS-2B cells after exposure to PM2.5 for 5 or 10 days. There were no obvious changes in p-JNK and p-ERK1/2 levels at either time point (data not shown), but PM2.5 exposure increased p-Akt protein level in a time- and dose-dependent manner. The change in p-Akt showed good agreement with that of DNMT3B protein at both time points. Moreover, pretreatment with LY294002, a PI3K inhibitor that can inhibit PI3K/Akt activation, almost abolished both the enhanced expression of phosphorylated Akt and DNMT3B up-regulation after 10 days of PM2.5 treatment (6 μg/cm^2^), and restored the expression of P53 (Figure 6E). In addition, the ROS scavenger, NAC also reversed these changes in p-Akt, DNMT3B, and P53 expression levels induced by repeated PM2.5 exposure. And the contamination from fibers did not have any significant effects on ROS generation and the signal pathway (Akt-DNMT3B-P53) we studied (Figure S1). In conclusion, our data strongly illuminates that ROS-PI3K/Akt pathway participates in the PM2.5-induced DNMT3B up-regulation, which leads to *P53* promoter hypermethylation and down-regulation of *P53* expression, a critical early event in epithelial cell carcinogenesis.

## DISCUSSION

A growing number of epidemiological studies have reported that PM2.5 exposure is associated with global and specific DNA methylation changes [11, 12, 32]. In this study, we demonstrate that repeated exposure to PM2.5 alters the methylation status of the *P53* promoter region via ROS-Akt-DNMT3B signaling pathway, subsequently resulting in reduced P53 expression in BEAS-2B cells. To our knowledge, our study is the first to identify the relationship between repeated exposure to PM2.5 and the methylation silencing of *P53* gene *in vitro*.

Numerous studies have assessed the toxicological effects of short-term exposure to PM, but the doses used in previous reports were always much higher than the actual exposure concentration [33, 34]. In one of our other studies (not published), we found that short-term, high-dose exposure to PM2.5 caused a completely different alteration in DNA methyltransferases expression compared with that after repeated exposure to PM2.5 at low concentrations. Here, we designed our doses based on the mass deposited on human tracheal and bronchial mucosa after a 24 h-exposure to 120 μg/m^3^ of PM2.5 using MPPD software, taking into account of the safety factor. After 10 days of exposure, the dose range enabled maintenance of cell growth but disrupted the regulation of growth-related genes and epigenetic modulators in BEAS-2B cells. Our results highlight the importance and significance of addressing the biological effects of repeated PM2.5 exposure at non-toxic doses.

After repeated exposure of BEAS-2B cells to PM2.5 (3 and 6 μg/cm^2^) for 5 days, the global DNA methylation level was dramatically decreased, consistent with previous studies [11, 35]. Three DNMTs participate in the maintenance of global DNA methylation pattern. In the present study, PM2.5 exposure decreased DNMT1 protein level, suggesting that PM2.5-induced down-regulation of DNMT1 may contribute to the reduction of global DNA methylation, although the signaling pathways involved remain to be explored. Hypomethylated DNA sequences in mammal cells are prone to transcriptional activation in response to environmental factors [36, 37], and long-term demethylation of many cytosine sites may result in genomic instability, gene mutation or aberrant expression of oncogenes [11, 38, 39]. Hence, we deem that genome-wide DNA hypomethylation may play a crucial role in lung carcinogenesis.

Meanwhile, we found that repeated PM2.5 treatment increased DNMT3B content in cells. As a critical enzyme participating in *de novo* DNA methylation, DNMT3B is thought to silence tumor suppress genes by methylating cytosine sites in gene promoters during the early stages of lung cancer development [21, 40]. Thus, we screened for genes silenced by PM2.5 exposure using PCR, and found that the relative expression of *P53* mRNA was significantly decreased after 10 days of repeated exposure to 6 μg/cm^2^ of PM2.5. When treated with DNMT3B-siRNA, the expression of P53 protein was restored to the normal level, strongly suggesting that the PM2.5-induced overexpression of DNMT3B participates in the transcriptional silencing of *P53*.

As reported, BEAS-2B cell line possesses normal P53 activity [41, 42], so it is a reliable model of researching P53 expression changes during PM exposure. P53, as a guardian of the genome, plays a critical role in biological processes and cellular homeostasis [13, 14, 43]. Down-regulation or mutation of *P53* are frequent features in different types of cancer, including lung cancer [44–46], and perturbation of *P53* promoter methylation may play an important role in its inactivation or mutation [15, 16, 47]. Therefore, we measured the methylation status of the *P53* gene promoter region by BSP after PM2.5 exposure and observed a dose-dependent increase in cytosine methylation. Surprisingly, however, the methylated sites were randomly distributed and almost non-CpG sites, and the number of methylated cytosines was markedly less than that reported in other studies. Previous studies reported that DNMT3B may interact with DNMT3A in catalyzing CpN methylation, which could provide a good explanation for the hypermethylation of non-CpG cytosine in the *P53* promoter in our study [48]. These methylated cytosine sites in the promoter may hinder binding of nuclear proteins to the sequence, resulting in silencing of *P53* gene expression [23, 29]. Indeed, online analysis revealed that there was a potential C/EBP binding site in the methylated sequence (−623 to −603 bp), with a methylated cytosine located at positon −618 bp, which was induced by 6 μg/cm^2^ of PM2.5. The EMSA test also verified the existence of the binding sites for TFs in this methylated region and reduced binding of TFs to the methylated sequences. So as a whole, our results strongly suggest that repeated exposure to PM2.5 suppresses *P53* transcriptional expression through hypermethylation of its promoter mediated by up-regulated DNMT3B protein. Methylation silencing of *P53* may be a critical event in the development of lung cancer induced by PM.

**Figure 7:**
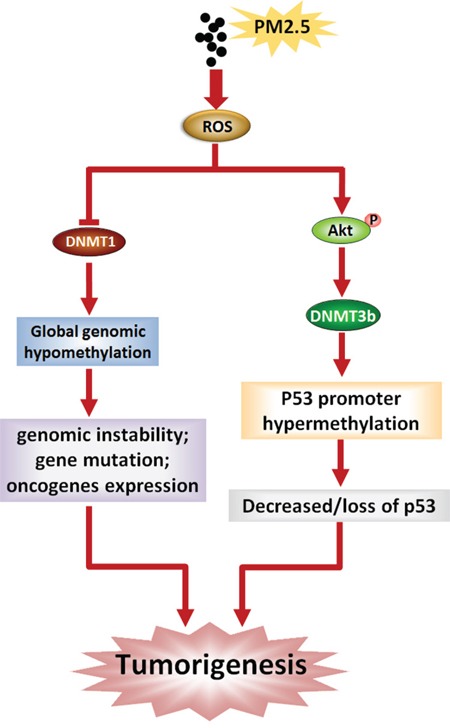
Schematic representation of lung carcinogenesis induced by PM2.5

Many different signaling pathways have been reported to participate in the regulation of DNMT3B [49, 50], but there is still no general consensus on which are necessary for DNMT3B regulation in cells exposed to PM. In present study, we observed continuous elevation of ROS and concomitant phospho-activation of Akt in BEAS-2B cells during exposure to PM2.5. These changes were blocked by pretreatment with the free-radical scavenger NAC, suggesting that PM2.5 activates Akt by increasing ROS. The Akt kinase regulates diverse biological processes [51, 52], and frequent hyperactivation or constitutively activation of Akt was found in some cancer cells [24, 53]. Akt kinase has also been reported to increase and/or stabilize DNMT3B content at transcriptional and post-transcriptional levels [22, 23]. We found that both LY294002 and NAC reversed DNMT3B up-regulation and P53 down-regulation induced by PM2.5 exposure, indicating that ROS-Akt-DNMT3B pathway participates in epigenetic silencing of special genes, notably *P53*, during the development and progression of PM-induced lung cancer. Based on the results mentioned above, a possible molecular mechanism of lung carcinogenesis induced by PM2.5 was presented in Figure 7.

In conclusion, exposure to PM2.5 at low concentrations induced no acute cytotoxicity of epithelial cells but led to chronic epigenetic changes associated with inhibition of *P53*, a common finding in lung cancer. These effects were attributed to the continuous activation of the ROS-Akt-DNMT3B pathway induced by PM2.5. Furthermore, PM2.5 also down-regulated global DNA methylation level, possibly by decreasing DNTM1 content. Therefore, our study not only provides experimental data for epidemiological association studies and a possible explanation for PM-induced lung cancer, but also may help to identify specific interventions to prevent PM-induced lung carcinogenesis.

## MATERIALS AND METHODS

### PM2.5 collection, extraction and characterization

PM2.5 samples were collected in Houguanhu Street, zhuankou economic development district, Wuhan city from April 14 to April 28, 2011. The PM2.5 sampling site is located in a residential area and more than 100 meters away from the main roads, without industrial sources of pollution nearby. PM2.5 was collected and extracted according to our previous report [54], then stored at −20°C. PM2.5 was re-suspended in Dulbecco's modified Eagle's medium (DMEM, Gibco) containing 2% fetal bovine serum (FBS) and sonicated for 30 min just prior to use. After sonication, the mean diameter and particle size distribution of PM2.5 was measured by a NANOPHOX instrument (Sympatec, German). Stability of PM2.5 in suspension was assessed by Turbiscan Lab^®^ Expert (Formulation, France) using a pulsed near-infrared LED at a wavelength of 880nm for 24 h. The PM2.5 suspensions were pipetted onto 300-mesh copper grids. After waiting 24 h to allow the particles to fall onto the grids, the shape of the particles was examined under a transmission electron microscope (TEM, Hitachi H-7650, Japan).

### Cell culture and exposure to PM2.5

The BEAS-2B human bronchial epithelial cell line was grown in DMEM supplemented with 10% FBS, 100 U/ml penicillin, and 100μg/ml streptomycin. The cells were maintained in a humidified incubator with 5% CO_2_ at 37°C. They were split and passaged every three days using 0.25% trypsin-EDTA. The cells were treated daily with fresh PM2.5 suspension for 10 consecutive days, with passage every 3 days. The exposure doses adopted in our study were designed by MPPD software (The Hamner Institutes for Health Sciences, USA) based on the real-world exposure scenarios. In some experiments, the cells were pre-incubated with N-acetylcysteine (NAC) (1mM, Sigma-Aldrich, Italy) or the Akt inhibitor LY294002 (50 μM, Cell Signal, USA) for 1 h prior to exposure to PM2.5.

### CCK-8 and LDH release assays

Cell viability was assessed by Cell counting Kit-8 (CCK-8) assay (Dojindo, Japan). Briefly, 5,000 BEAS-2B cells per well were seeded in 96-well plates and exposed to different concentrations of PM2.5 for 24 h or 10 days. Then the PM2.5 suspension was replaced with an equal volume of fresh medium containing 10% CCK8, followed by 3-h incubation at 37°C. The absorbance was determined at 450 nm on a micro-plate reader (Multiskan MK3, Thermo Fisher Scientific, USA). The lactate dehydrogenase (LDH) release in the medium was measured to estimate the extent of cell damage. Briefly, culture medium was centrifuged at 1000 rpm for 5 min and then incubated with the prepared reagent mixture at room temperature for 20 min according to the manufacturer's protocol (Applygen Technologies, China). The absorbance of samples was determined at 450 nm on a micro-plate reader.

### Western blot

BEAS-2B cells were lysed in cold RIPA buffer with protease and phosphatase inhibitors (Applygen Technologies), followed by standard protein extraction procedures. Protein concentration was then measured with a BCA protein assay kit (Applygen Technologies). Protein samples were denatured at 99°C for 5 min, separated on 10% SDS-PAGE gels (45 μg/lane), and transferred to PVDF membranes (Millipore, USA). Membranes were blocked with 5% skim milk in Tris-buffered saline plus Tween 20 (TBST) overnight at 4°C and then incubated separately with a primary antibody against P53, phospho-P53 Ser15, DNMT1, DNMT3A, DNMT3B (1:1000, Abcam), β-actin (1:3000, Abcam), Akt or phospho-Akt Ser473 (1:1000, Cell Signal). After 3 washes with TBST (10 min each), the membranes were incubated with an appropriate secondary antibody for 1 h at room temperature (anti-rabbit IgG, Abcam, 1:3000; anti-mouse IgG, Abcam, 1:3000), followed by washing and incubation with SuperECL Plus detection reagents (Applygen Technologies), and then exposed to film. Densitometric analysis of bands was performed using Image J software. Band intensities were normalized to that of β-actin and expressed as fold-change over control.

### Global DNA methylation quantification and bisulfite sequencing PCR (BSP)

Genomic DNA was extracted using the TIANamp Genomic DNA Kit (TIANGEN BIOTECH, China). Global DNA methylation levels were detected using Methylamp™ Global DNA Methylation Quantification Ultra kit (Epigentek, USA). Absorbance values at 450 nm were measured in a micro-plate reader and the results were expressed relative to the control. For BSP analysis, genomic DNA was bisulfite-treated according to the EZ DNA Methylation-Gold™ Kit instruction manual (ZYMO RESEARCH, USA). The *P53* promoter sequence was amplified from the converted DNA by touch-down PCR, and then the purified PCR products were cloned into pMD-18T vector (Takara, China) for sequencing. For each group, 10 individual clones were sequenced to identify the methylated cytosine bases. Prediction of the CpG islands and designation of BSP primers for *P53* promoter region (−979 to +243 bp) were performed on the Methprimer website (http://www.urogene.org/methprimer/). PCR conditions and BSP primer sequences for CpG islands were shown in supplementary material (Table S2).

### Electrophoretic mobility shift assay

Nuclear protein was extracted with the Nuclear and Cytoplasmic Protein Extraction Kit (Beyotime, China) and stored at −80°C. The electrophoretic mobility shift assay (EMSA) was performed with the Light Shift^®^ Chemiluminescent EMSA Kit (Thermo Fisher scientific) according to the manufacturer's instructions. Briefly, DNA probes were synthesized according to the predicted binding site in the *P53* promoter (−623 to −603 bp) and labeled with biotin (Probes sequences were shown in Table S2). The probes were denatured at 95°C for 5 min and annealed to double-stranded DNA probes in a water bath. The binding reaction mixture, with or without 3 μl of nuclear proteins, was separated on a 5% native PAGE gel. The proteins were transferred onto 8 × 6 cm positively charged nylon membrane, then immediately UV cross-linked for 45 s in a Benchtop 3UV™ Transilluminator with 254nm wavelength, followed by detection using horseradish peroxidase (HRP) conjugated streptavidin and chemiluminescent substrate.

### Quantitative real time PCR

Total RNA was isolated from BEAS-2B cells with TRIzol® reagent (Invitrogen, USA), and then reverse transcribed into cDNA using the RevertAid™ First Strand cDNA Synthesis kit (Thermo Fisher Scientific) according to the manufacturer's instructions. Primers for *P53* and β-actin were designed using Primer Premier 5 software and synthesized by SBS Genetech (Beijing, China). Primer sequences for *P53* and β-actin are listed in Table S2. PCR amplification was performed on a Bio-Rad iQ™5 real-time PCR detection system using SYBR® Premix Ex Taq™ kit (Takara). Changes in *P53* expression were determined by normalizing to the control.

### siRNA transfection

BEAS-2B cells (2 × 10^5^/well) were plated in growth medium without antibiotics in 6-well plates. After 24 h, cells were transfected with 30 nM DNMT3B siRNA (sc-37759, Santa Cruz Biotechnology, USA) or control siRNA (sc-37007, Santa Cruz Biotechnology) using Lipofectamine® RNAiMAX Reagent (Invitrogen). The medium was refreshed after 5 h with growth medium. The transfection efficiency of DNMT3B siRNA was assessed by RT-PCR. Primer sequences are listed in Table S2. The cells were transfected with DNMT3B or control siRNA on day 4 and day 7 of the 10-day exposure to PM2.5. The Results were expressed relative to the control.

### ROS detection

BEAS-2B cells (2 × 10^5^) were seeded into 35-mm dishes and exposed to PM2.5. After 12 h of exposure to PM2.5 at different concentrations (1.5, 3, 6 μg/cm^2^) or 6 h, 12 h, 24 h, 48 h or 10 days of exposure to 6 μg/cm^2^ of PM2.5, cells were harvested for intracellular ROS detection using 5-(and-6)-chloromethyl-2′,7′-dichlorodihydrofluoresceindiacetate, acetyl ester (CM-H_2_DCFDA, Invitrogen). Briefly, the cells were washed twice with PBS and resuspended in serum-free medium containing 10 μM of CM-H_2_DCFDA. The cell suspensions were incubated at 37°C for 40 min in the dark and then rinsed three times with PBS to remove excess CM-H_2_DCFH-DA. Finally, cells were resuspended in 400 μl PBS and immediately analysed by a FACSCalibur flow cytometer for ROS detection (Becton Dickinson, USA).

### Statistical analysis

All results represent at least 3 independent experiments and are expressed as mean ± SD. Multiple group means were first compared by one-way ANOVA followed by LSD test for pairwise comparisons. All statistical analyses were conducted using SPSS software (SPSS program, version 13.0, Chicago, IL, USA). *P < 0.05* was considered statistically significant.

## SUPPLEMENTARY FIGURE AND TABLES


