# Supplementary material for: Repeated PM2.5 exposure inhibits BEAS-2B cell P53 expression through ROS-Akt-DNMT3B pathway-mediated promoter hypermethylation
Source: Oncotarget. 2016 Mar 2;7(15):20691–703. doi: 10.18632/oncotarget.7842 (PMC4991485; doi:10.18632/oncotarget.7842)
Supplement: Supplementary file 1 [file oncotarget-07-20691-s001.pdf]

SUPPLEMENTARY FIGURE AND TABLES

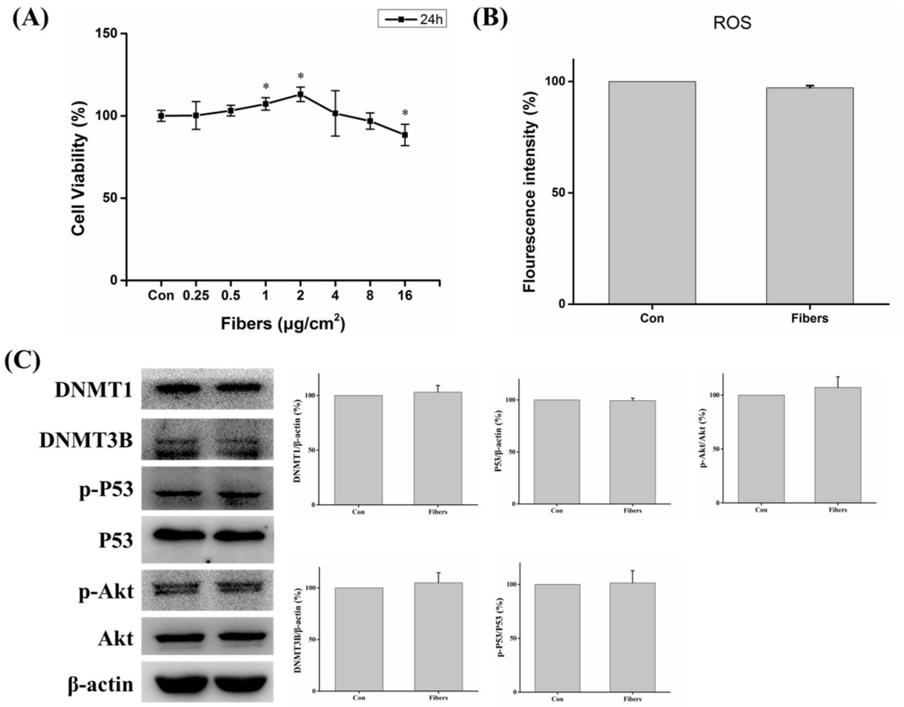

Supplementary Figure S1: Fibers released from glass filters did not exert any effects on cell viability and the ROS-Akt-DNMT3B-P53 signal pathway. A, B. BEAS-2B cells were treated with fibers (0.247 μg/cm^2) for 24 h, cell viability and intracellular ROS production were measured by CCK-8 and Flow cytometry, respectively. **C.** Cells were exposed to 0.247 μg/cm^2 of fibers for 10 days, protein lysates were immunoblotted with indicated antibodies, and quantification of protein bands relative to β-actin using the Image J software.

Supplementary Table S1: MPPD baseline settings

MPPD baseline input categories	Baseline input settings
Individual characteristics (airway morphometry and deposition/ clearance)	Human species; Yeh-Schum symmetric single path lung model; FRC = 2950 mL; URT volume = 50 mL; Tracheal mucous velocity = 5.5 mm/ min; fast human clearance rate = 0.02/day; medium human clearance rate = 0.001/ day; slow human clearance rate = 0.0001/day; lymph node human clearance rate = 0.00002/day
Exposure scenario: constant exposure	Acceleration of gravity = 981.0 cm/sec^2; body orientation = upright; aerosol concentration = 0.12 mg/m^3; breathing frequency = 20/min; V_T= 700 mL; inspiratory fraction = 0.5; pause fraction = 0; breathing scenario, nasal breather; number of hours per day = 24; number of days per week = 1; number of weeks = 1; maximum postexposure days = 0
Particle properties	Density = 1 g/cm^3; diameter = 0.739 µm; count median diameter checked; Nanoparticles model not checked; inhalability adjustment not checked; GSD (diameter) = 1.195

Abbreviations: FRC, functional residual capacity; V_T, tidal volume; GSD, geometric standard deviation

Supplementary Table S2: Primers, DNA probe sequences and conditions for RT-PCR, BSP or EMSA

See Supplementary File S1
